# Acoustic Performance of Floors Made of Composite Panels

**DOI:** 10.3390/ma16052128

**Published:** 2023-03-06

**Authors:** Jacek Nurzyński, Łukasz Nowotny

**Affiliations:** Instytut Techniki Budowlanej, 00-611 Warsaw, Poland

**Keywords:** sound insulation, lightweight buildings, Fiber Reinforced Polymers, floors, composite panels

## Abstract

Airborne and impact sound insulation of composite panels arranged in different configurations were investigated in this study. The use of Fiber Reinforced Polymers (FRPs) in the building industry is growing; however, poor acoustic performance is a critical issue for their general employment in residential buildings. The study aimed to investigate possible methods of improvement. The principal research question involved the development of a composite floor satisfying acoustic expectations in dwellings. The study was based on the results of laboratory measurements. The airborne sound insulation of single panels was too low to meet any requirements. The double structure improved the sound insulation radically at middle and high frequencies but the single number values were still not satisfactory. Finally, the panel equipped with the suspended ceiling and floating screed achieved adequate level of performance. Regarding impact sound insulation, the lightweight floor coverings were ineffective and they even enhanced sound transmission in the middle frequency range. Heavy floating screeds behaved much better but the improvement was too small to satisfy acoustic requirements in residential buildings. The composite floor with a dry floating screed and a suspended ceiling appeared satisfactory with respect to airborne and impact sound insulation; the single number values were R_w_ (C; C_tr_) = 61 (−2; −7) dB, and L_n,w_ = 49 dB, respectively. The results and conclusions outline directions for further development of an effective floor structure.

## 1. Introduction

Lightweight buildings are perceived as environmentally friendly, consistent with the sustainable development concept and providing good interior quality [1]. They are beneficial with respect to construction, building physics and energy conservation. In effect, the marked trend of lightweight residential buildings is evidently growing and the tendency toward industrialization in the construction sector causes panelized and modular homes to become increasingly popular. The buildings are usually supported with wood or metal frames and equipped with faces constructed of various thin panels, e.g., oriented strand boards (OSB), plasterboards, gypsum fiberboards, fiber-cement boards and cement-bonded particle-board. Adequate sound insulation and thermal resistance are provided by additional insulating layers [2]. In recent times considerable efforts have been taken to involve composite panels with polymeric faces in lightweight building structures; their acoustic performance, however, remains a critical issue for their general employment in housing [3,4].

Fiber Reinforced Polymers (FRPs) are widely employed in different industries for a great variety of products [5,6]. Due to their properties such as light weight, high strength, excellent corrosion and fatigue resistance and convenient installation FRP composites are widely used for renovation, repair and structural reinforcement [7,8]. Thus far, civil engineering applications have focused mainly on objects like bridges or footbridges [9,10]. The types of FRPs mainly include carbon fiber reinforced polymers (CFRP), glass fiber reinforced polymers (GFRP) and basalt fiber reinforced polymer composites (BFRP) [11]. The durability and mechanical properties of CFRP are better than GFRP and BFRP but the high cost of carbon fiber may limit some of their applications [12], unlike, for example, GFRP, which is characterized by rich sources and a low cost of production. The combination of two or three types of fibers in FRP can be made to achieve high durability and high strength at relatively low cost, taking advantage of the corrosion resistance of carbon fiber and the low price of basalt and glass fibers [13]. The use of natural fibers in polymer composites increases to meet a number of end-use applications in transportation, geotextiles and low-cost constructions [14]. They have relatively low mechanical properties but play an important role in the development of biodegradable materials to replace glass and carbon fibers and plastics reinforced with inorganic fillers [15,16]. When taking into account growing concerns about global warming and rising prices of petroleum products, such materials have good prospects [17,18]. One of the disadvantages of a natural fiber composite is relatively high water-absorbing behavior when compared with synthetic fiber equivalents. However, the addition of Polypropylene-grafted maleic anhydride lowers the moisture absorption rate of WPC (Wood Plastic Composite) [19].

Composite panels have been employed for a long time in the automotive, ship and aerospace industries due to the possibility of replacing aluminum materials with GFRP cores and FRP cladding without affecting the acoustic properties of the panel while reducing the weight [20]. However, their acoustic performance publications are rather scarce [21]. The structure of panels with polymeric faces is similar to typical sandwich panels consisting of an insulating core and metal faces, which are widely used in industrial or storage buildings. Thus, it may be expected that their sound insulation characteristics are also similar. Due to the sandwich structure, the mass of faces resonates against the stiffness of a core and brings about local enhancement of sound transmission in a certain frequency range. The fundamental mass–spring–mass resonance may be approximated based on a core’s dynamic stiffness and the face’s surface mass [22]. The sound transmission loss of partitions consisting of thin panels is also influenced by coincidence; this occurs when the speed of bending wave propagation is equal to the acoustic wave speed in the surrounding medium. In glazing or plasterboard frame walls, this usually appears at middle or high frequencies depending on the plate thickness and its surface mass. The structure of sandwich panels, however, is somewhat different. The critical frequency of faces themselves is above the interest range, but the coincidence occurs for any panel capable of sustaining shear stress, so it also exists for the entire cross-section and should be expected at low frequencies due to the low mass and relatively high rigidity.

Several specific analytical models were developed for modelling the acoustic behavior of sandwich panels [23,24], and a thorough review in this field was conducted by D’Alessandro et al. [25]. In addition, numerous general prediction models exist for double panel structures based on the classical impedance approach [26], progressive-wave theory [27], transfer matrix methods [28], spatial windowing technique [29], statistic energy analysis [30] or effective medium method (EM) [31,32]. An extensive study of double-wall prediction methods was elaborated by Hongisto [33]. However, analytical expressions do not seem to agree well with the measured results.

Available publications specifically dedicated to the acoustic performance of composite panels are very limited. Patinha et al. conducted acoustic tests on samples with dimensions of 200 mm × 200 mm. However, an untypical test facility was used for the research and it is difficult to compare the results with others [34]. There is only one literature position reporting experimental results for a composite floor; only one single panel without any additional layers was investigated. The work focused on numerical simulation of the acoustic parameters of a bare floor slab using the Finite Element Method (FEM) [35]. No improvement methods for the airborne and impact sound insulation of composite floors have been investigated before; no such results are reported in the literature.

The lack of experimental data and the inaccuracy of analytical models were the main reasons for conducting comprehensive experimental research on the acoustic behavior of the composite panels. This study aimed mostly to investigate the sound insulation of floor prototypes equipped with different additional insulating layers. The double set-ups arranged in different configurations, suspended ceilings and floating screeds were examined. The principal research question involved the development of a floor satisfying acoustic requirements in dwellings.

## 2. Experimental Procedures

### 2.1. Test Procedure

The samples were installed in test facilities with suppressed flanking transmission complying with the requirements of PN-EN ISO 140-1:1999. The test facility for small samples consisted of two rooms: the source room had a volume of 87.5 m^3^ and receiving room was 51.6 m^3^. The sample was mounted in a test opening (adapted to the dimensions of the sample) in a double wall with the following structure: a wall made of silicate blocks 240 mm thick + mineral wool 30 mm thick (in the axis between the chambers of the test stand) + a wall made of silicate blocks 180 mm thick.

The full-scale models were tested in a horizontal test opening intended for floors. Airborne and impact sound insulation were carried out on samples mounted in the same conditions. Both the source (100 m^3^) and receiving (90 m^3^) rooms were of irregular shape with no parallel walls and were separated by a structural acoustic break.

Airborne and impact sound insulation tests were performed per EN ISO 10140. Single number quantities were calculated according to EN ISO 717-1 and EN ISO 717-2, airborne and impact sound insulation, respectively. Two wideband speaker cabinets placed in the corners of the sending room were used as a pink noise source used for airborne measurements. The standard tapping machine (according to EN ISO 10140-5) placed at five different positions on the floor (in accordance with the standard’s recommendations) was used to determine the impact sound insulation. Average sound pressure levels in 1/3 octave bands were simultaneously measured in the source and the receiving room. Continuously moving microphones were used for the space averaging; the sound pressure level was integrated over time and space. Reverberation time in the receiving room was determined to enable the calculation of sound reduction index R and the normalized impact sound pressure level, L_n_.

### 2.2. Samples and Materials

The composite panels consisted of a core made of polyurethane foam and faces made of epoxy resin reinforced with glass fiber (GFRP). Small samples, 1390 mm × 2450 mm, and full-scale models, 4190 mm × 2720 mm, were investigated. The dimensions of the samples were in accordance with the recommendations of EN ISO 10140. Four types of small panels were examined: m1, m2, m3 and m4. The panels differed with a thickness of 35 mm and 60 mm, and a core density of 40 kg/m^3^ and 70 kg/m^3^. The core of the m4 panel was separated from the face on one side with an interlayer made of elastic EPDM (ethylene propylene diene monomer), 4 mm thick. Large models were denoted by: M1, M2 and M3; they were constructed of three smaller elements bound together. Basic technical data on the samples are presented in Table 1. The thickness and weight of individual elements of the same type were not exactly the same; Table 1 contains mean values.

**Figure 1:**
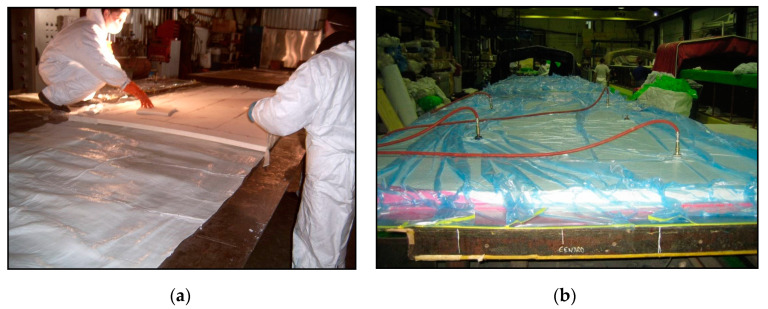
(**a**) Laying pre-pregs with polyurethane foam; (**b**) Using vacuum pump technology to consolidate panel layers (photo Mostostal Warszawa).

The elements were manually manufactured by Mostostal Warszawa using the so-called pre-impregnates (pre-preg). The production method was based on the use of previously prepared glass fabrics saturated with epoxy resin. The next step was the arrangement of pre-impregnates and polyurethane foam and shaping the element. Then began the consolidation of the layers of the composite panel; vacuum bagging technology was used for this process. The last stage of production was heating the element. Figure 1 illustrates the process of samples preparation.

This method of production was largely manual and labor-intensive, but it did not require high investments related to the start-up of production. Furthermore, the advantages of this technology were simplicity and low initial manufacturing costs which contributed to the choice of this production method.

Standalone panels, double set-ups and several arrangements of M1 panels with additional insulating layers, i.e., suspended ceilings, floating screeds and floor coverings, were considered. Double set-ups consisted of two panels installed at a distance of 30 mm and 100 mm; the cavity between them was empty or filled with mineral wool. Denotations of double set-ups contain symbols of both panels, the distance between them and information on mineral wool in the cavity; e.g., (m1-30 MW-m1) means two m1 panels at a distance of 30 mm with mineral wool (MW) in the cavity. In theory, in the range above the fundamental mass–spring–mass resonance the sound insulation of double panels separated by a cavity is much higher than an equivalent weight single panel. This is due to the damping mechanism of the air space which couples both panels. The resonance effects emerging in the cavity may be mitigated by installing a sound absorbing material [36].

**Figure 2:**
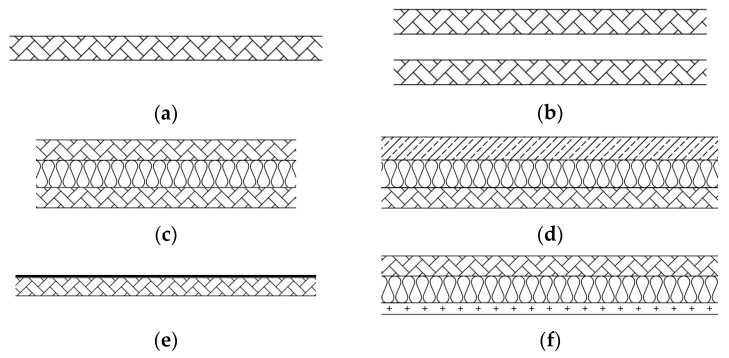
Schemes of tested arrangements (**a**) single panel; (**b**) double set-up with empty cavity; (**c**) double set-up with mineral wool; (**d**) panel with a floating floor; (**e**) panel with a lightweight floor covering; (**f**) panel with a suspended ceiling.

The suspended ceiling was made of single 12.5 mm (C1x) and double 2 mm × 12.5 mm (C2x) plasterboards installed at a distance of 150 mm beneath the M1 panel; the cavity was filled with 150 mm of mineral wool. Floor coverings consisted of typical floor panels (FP), 7 mm thick, and resilient underlayers made of corkboard 3 mm (cb.3 mm), corrugated paper board 3 mm (cpb.3 mm), polyester foam 5.5 mm (pef.5.5 mm), and polyethene foam 3 mm (ptf.3 mm). Heavy floating screeds consisted of a cement plate (CP), 40 mm thick, positioned successively on: elastic polystyrene E-EPS 17/15, E-EPS 33/30, E-EPS 43/40 and mineral wool (MW) 30 mm. Lightweight prefabricated dry floating screed consisted of a resilient underlayer made of mineral wool, 15 mm thick bound with the top layer of double 2 mm × 12.5 mm gypsum fiberboards. Figure 2 presents schemes of investigated arrangements.

## 3. Airborne Sound Insulation, Results of Measurements and Discussion

### 3.1. Initial Survey Based on Small Elements

#### 3.1.1. Standalone Panels

Small samples, 1390 mm × 2450 mm, were initially tested to explore possible acoustic tendencies resulting from the panels’ basic structure and gain some guidelines for further research involving full-scale models. Small elements may be used in practice as complementary, filling members in the frame (beam) floor; hence the results are also interesting in this respect.

**Figure 3:**
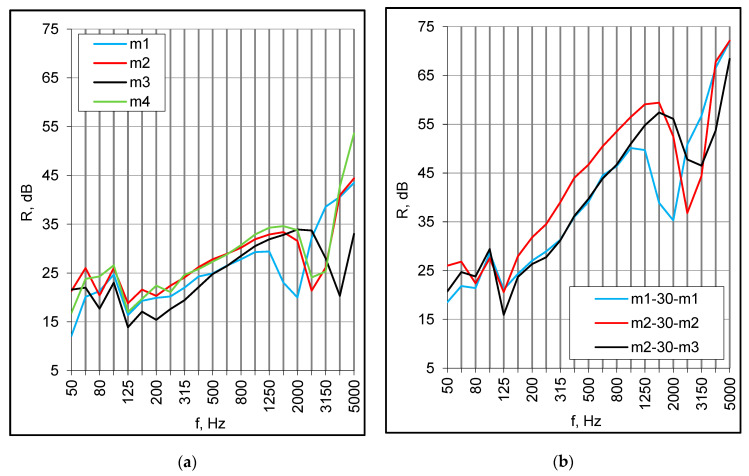
(**a**) Sound reduction index of standalone panels, small samples; (**b**) Sound reduction index of double set-ups with 30 mm empty cavity, small samples.

Sound insulation of standalone panels, as expected, was similar to typical sandwich panels consisting of an insulating core and faces made of corrugated steel sheets. The high-frequency resonance occurred at 2000–4000 Hz depending on the panel thickness, core density and surface mass of faces (Figure 3a). For lightweight partitions composed of thin plates, the decrease at high frequencies is usually related to the coincidence. However, the structure of sandwich panels is specific; they have relatively high bending stiffness and low surface mass. Thus, the coincidence of the entire panel should be expected at low frequencies [37], and a pronounced lowering of sound insulation was observed at 200 Hz (Figure 1a). The fundamental resonance occurred at 125 Hz. Single number values were quite the same for all samples regardless of their structure; the thickest panel had lower sound insulation expressed by the (R_w_ + C_tr_) index (Table 2). The values were influenced mainly by the sound insulation at middle frequencies, which basically depended on the surface mass of faces, while the parameters of the core were less important.

#### 3.1.2. Double Set-Ups

Double set-ups consisted of two panels arranged with a distance of 30 mm and 100 mm between them. The panels were installed on either side of the vibration break of the test facility, so they were entirely separated from each other. The first series of measurements were taken for the arrangements with a 30 mm empty cavity, i.e., the plenum between panels was not filled with any absorbing material. Two symmetrical configurations (m1-30-m1 and m2-30-m2) and one asymmetrical (m2-30-m3) were investigated.

The double structure improved the sound insulation radically at middle and high frequencies (Figure 3b). The values of the R_w_ + C index were within the range of 38–41 dB (Table 3); they increased by several decibels in comparison with standalone panels, but still were too low to satisfy any requirements for floors between dwellings. For symmetrical double set-ups, the high-frequency resonance was more pronounced than for single panels. For asymmetrical double set-ups, this effect was mitigated due to the different positions of the individual resonances (Figure 3b), but the local improvement did not significantly influence the single number values because of the low and middle frequency behavior.

**Figure 4:**
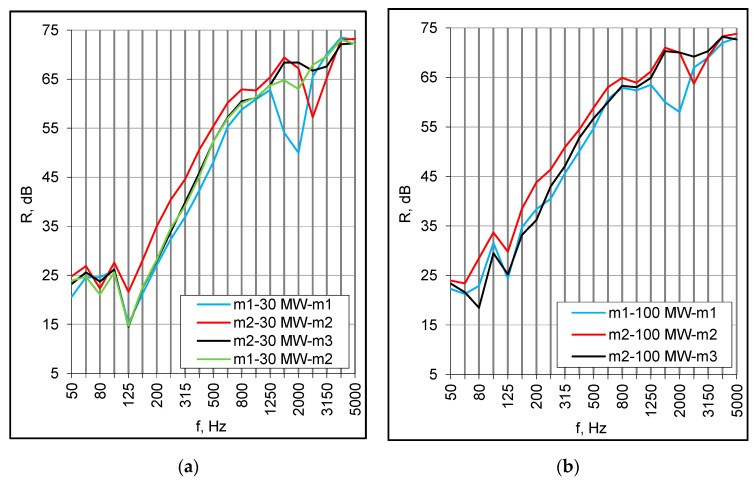
(**a**) Sound reduction index of double set-ups with 30 mm cavity filled with mineral wool (MW), small samples; (**b**) Sound insulation of double set-ups with 100 mm cavity filled with mineral wool (MW), small samples.

**Figure 5:**
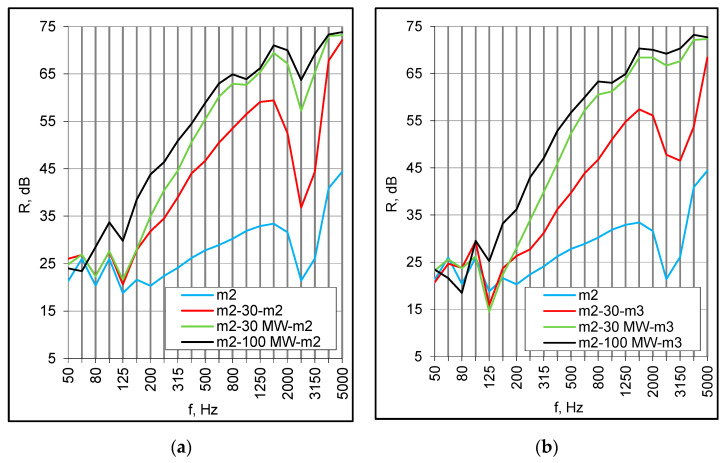
(**a**) Sound reduction index of m2 panels in different configurations, small samples; (**b**) Sound insulation of m2/m3 panels in different configurations, small samples.

The second series of measurements were taken for the same set of panels but with the plenum filled with 30 mm of mineral wool. The absorption caused the high-frequency dip to be smoother and almost totally eliminated for asymmetrical set-ups (Figure 4a,b). Finally, the same combinations of panels were installed at a distance of 100 mm and the plenum was filled with mineral wool. The values of the R_w_ + C index increased considerably and ranged from 48 dB to 54 dB, which gives a reasonable prospect for double floors in modular buildings (Table 3). The low-frequency resonance occurred at 125 Hz regardless of the panels’ structure and arrangement (Figure 5a,b).

### 3.2. Full-Scale Models

#### 3.2.1. Single Panels

**Figure 6:**
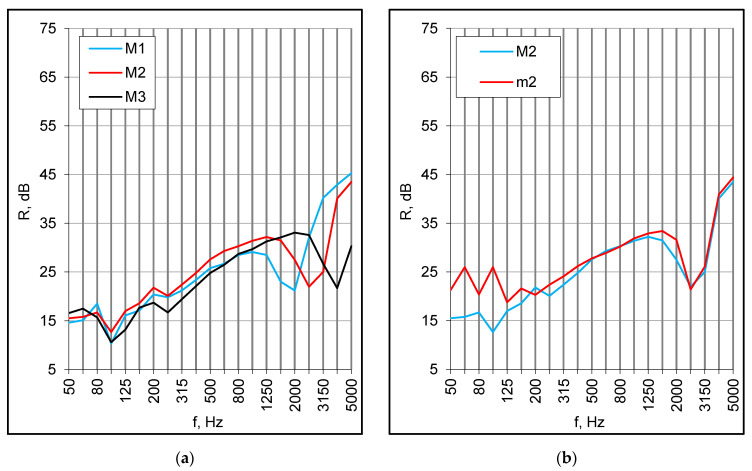
Sound reduction index of standalone panels, (**a**) full-scale models; (**b**) comparison of small elements and full-scale models.

Full-scale models, 2720 mm × 4190 mm, were tested in a horizontal test facility designed for floors, roofs and ceilings. The sound insulation characteristics of standalone panels M1, M2 and M3 were broadly similar; differing basically at high frequencies due to the position of the resonance (Figure 6a). Surprisingly, except for the low-frequency area, the characteristics were consistent with respective plots obtained previously for small elements (Figure 6b). The differences resulted from the position of fundamental resonance, whereas for the full-scale models, this moved down by 1/3 octave and caused a significant reduction of the sound transmission loss. The differences, however, had little influence on the single number values, and the respective indices for small samples and full-scale models were nearly the same (Table 2).

#### 3.2.2. Double Set-Ups

**Figure 7:**
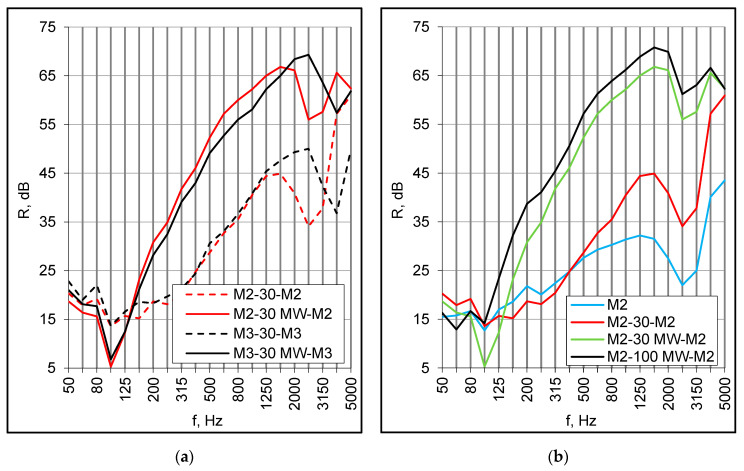
(**a**) Sound reduction index of double composite panels, the effect of mineral wool in the cavity; (**b**) Sound reduction index of M2 panels in different configurations, full-scale models.

Double set-ups were of the same configuration as corresponding small samples, i.e., they consisted of two panels with an empty cavity and a cavity filled with mineral wool. The samples were constructed of two M2 and two M3 panels arranged symmetrically at a distance of 30 mm and 100 mm. The results for both panels were similar except for the area of a high-frequency resonance (Figure 7a). In both set-ups with an empty cavity, due to the low frequency resonance, the values of the R_w_ + C index were merely 4 dB higher than for single componential panels (Table 2 and Table 3).

The mineral wool in the cavity brought about further improvement at middle and high frequencies. Consequently, the R_w_ index increased by several decibels, but at the same time, the negative values of C and C_tr_ terms decreased due to the low-frequency resonance. Unexpectedly, this was more prominent for samples with 30 mm of absorption than for any other configuration, including single M2 and M3 panels (Figure 7b). Usually, for symmetrical double structures, the low-frequency drop is more pronounced for samples without absorption in the cavity. In effect, the R_w_ + C_tr_ index was lower than for the respective double set-up with no absorption. The arrangements consisting of M2 panels and mineral wool produced slightly better results in the middle frequency range, but due to the low-frequency behavior, the R_w_ + C index values were the same as for M3 configurations (Table 3).

**Figure 8:**
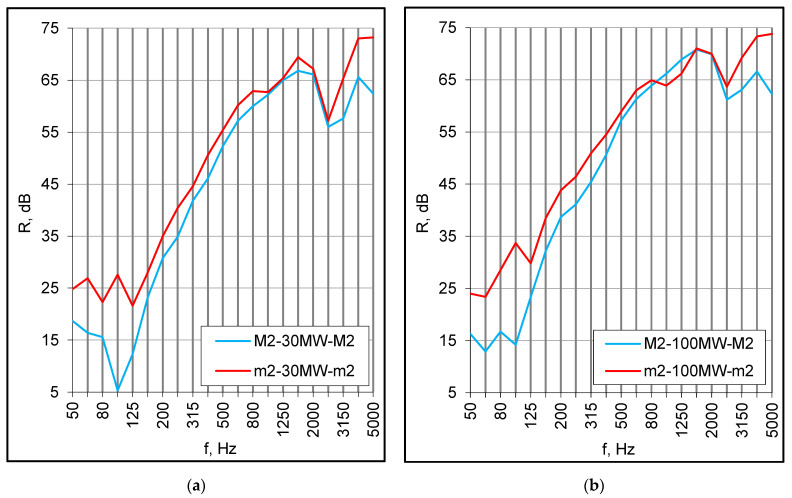
(**a**) Double set-ups with 30 mm of mineral wool (MW), sound reduction index of small elements and full-scale models; (**b**) Double set-ups with 100 mm of mineral wool (MW), sound reduction index of small elements and full-scale models.

The double set-ups behaved somewhat differently than single panels. The low-frequency distinctions between small and large samples were more prominent and ranged from 20 to 23 dB at 100 Hz (Figure 8a,b). They were related to the panels’ dimensions and their modal behavior, which also influenced the middle-frequency results to a certain degree, in consequence of the fundamental resonance lowering. In effect, the R_w_ + C index values for full-scale models were 11–12 dB lower than for corresponding small samples (Table 3).

**Figure 9:**
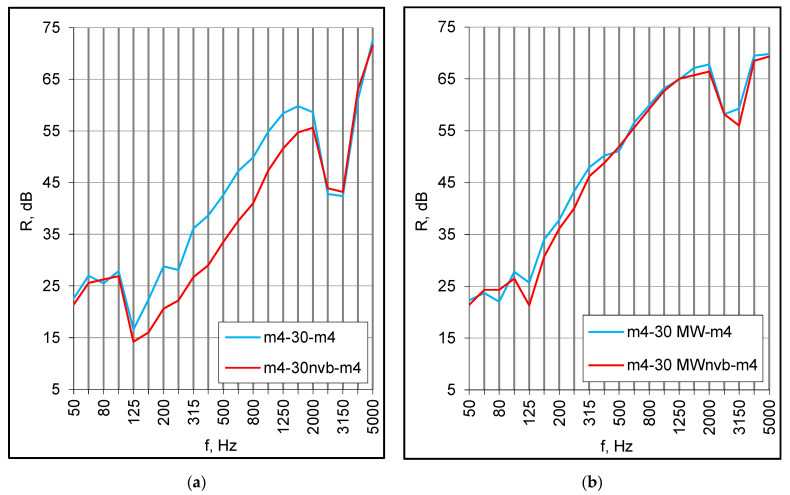
(**a**) The effect of an absence of a vibration break (nvb), double set-ups with an empty cavity; (**b**) The effect of an absence of a vibration break (nvb), double set-ups with mineral wool (MW).

Another possible reason for the middle frequency discrepancies was the supporting conditions. Small panels were installed in a heavy double filler wall on either side of the vibration break of the test facility, so they were totally separated from each other. In turn, the panels of full-scale models were connected on the perimeter with wooden battens, creating a structural path of sound transmission. The effect of such a connection may be observed when comparing the results obtained for the same combinations of small panels but installed differently, i.e., on either side and on the same side of the vibration break (nvb). For double set-ups without absorption, the differences at middle frequencies reached several decibels (Figure 9a). The mineral wool mitigated this effect (Figure 9b), and in the case of a 100 mm cavity with absorption, it was barely noticeable.

**Figure 10:**
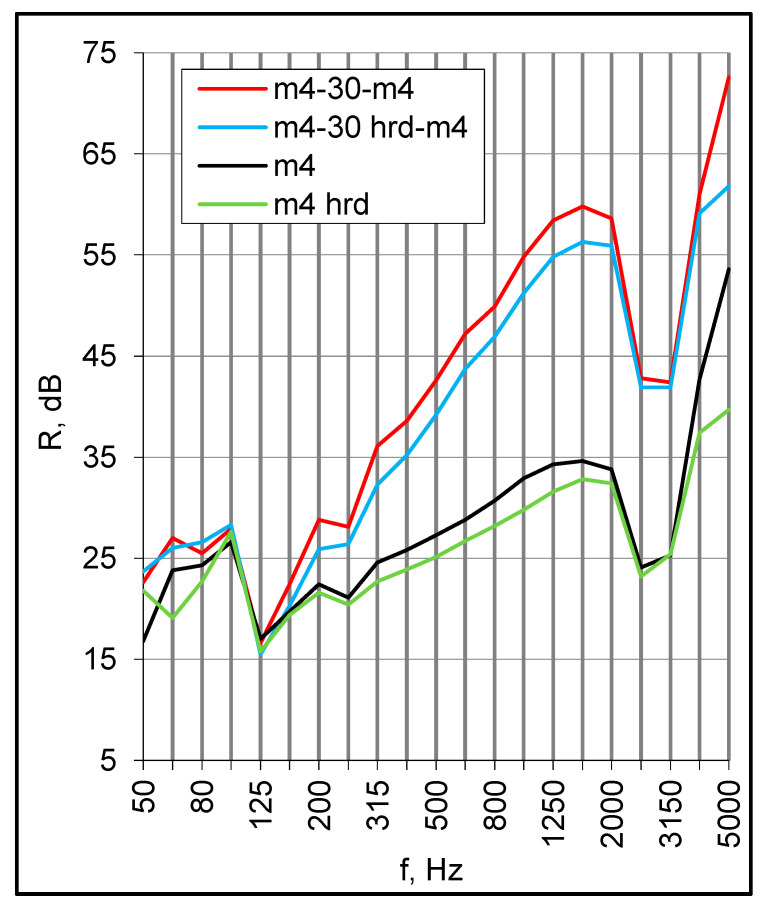
The effect of the panel edges support hardening (hrd) on the sound insulation.

The sound insulation at middle frequencies might also be reduced due to the edge clamping. Figure 10 illustrates the result of hardening (hrd) of the putty applied to the perimeter of the small samples to caulk them in the test opening. The sound insulation decreased by 2–3 dB in this range, and the decrease was observed for the standalone panels as well as double set-ups. Similar reductions occurred when the panel edges were wedged in several points against the test opening frame.

#### 3.2.3. Additional Insulating Layers

**Figure 11:**
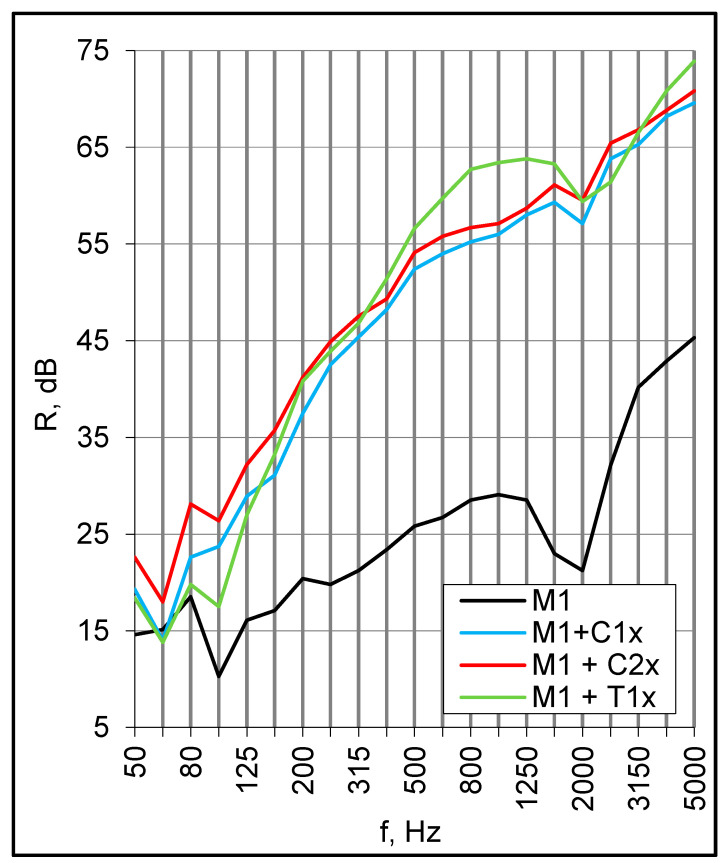
Sound reduction index of M1 panel with a suspended ceiling and top insulation.

The preceding results demonstrated that double set-ups’ capability for further improvement is somewhat limited. In search of another, more effective solution, the single M1 panel was equipped with a suspended ceiling. This was made of 1 mm × 12.5 mm (C1x) and 2 mm × 12.5 mm (C2x) plasterboards installed at a distance of 150 mm beneath the panel; the cavity was filled with 150 mm of mineral wool. In effect, the sound insulation increased greatly in almost the entire frequency range (Figure 11). The high-frequency resonance of the basic M1 panel was marked at 2000 Hz for all arrangements, whereas the coincidence of plasterboards was around 3150 Hz. In the next step, the suspended ceiling was substituted with a lining placed directly on the top of the M1 panel simulating the lightweight dry floating screed. This consisted of 100 mm of mineral wool and 15 mm plasterboards put freely on top (T1x). The effect was generally similar to the suspended ceiling; the low frequency lowering at 100 Hz was probably due to the structural path of sound transmission through the mineral wool supporting the upper plasterboard (Figure 11). Single number quantities for all samples are presented in Table 4. The arrangement with a double suspended ceiling seems promising; however, taking into consideration flanking transmission in a building, the R_w_ + C index was still too low to satisfy acoustic expectations in dwellings.

## 4. Impact Sound Insulation, Results of Measurements and Discussion

### 4.1. Initial Study Based on Small Samples of Flooring

Initially, the M1 panel (full scale) was tested with small samples of floating screeds and lightweight floor coverings (1000 mm × 1000 mm). The measurements were taken to get some experience on the composite panel’s behavior when impact excited and provide guidelines for further investigation based on full-scale models.

**Figure 12:**
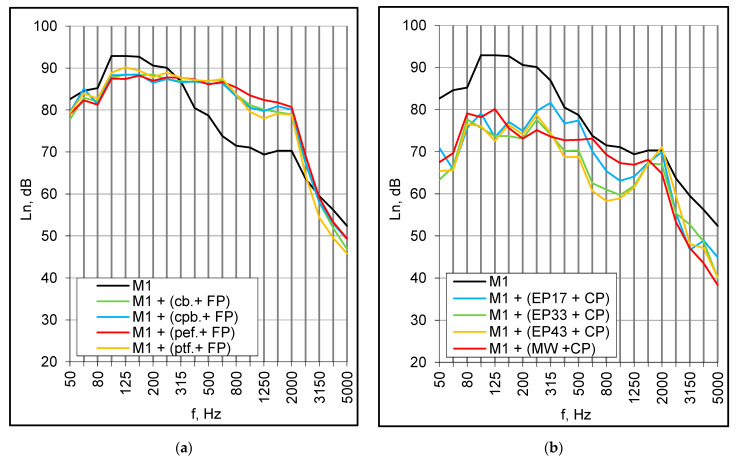
(**a**) M1 panel with floor coverings; floor panels (FP), corkboard, 3 mm (cb.3 mm), corrugated paper board, 3 mm (cpb.3 mm), polyester foam, 5.5 mm (pef.5.5 mm), polyethene foam, 3 mm (ptf.3 mm); (**b**) M1 panel with floating screed: cement plate, 40 mm (CP), E-EPS 17/15 (EP17), E-EPS 33/30 (EP33), E-EPS 43/40 (EP43), mineral wool (MW).

Floor coverings consisted of typical floor panels and resilient underlayers made of corkboard (3 mm), corrugated paper board (3 mm), polyester foam (5.5 mm) and polythene foam (3 mm). Generally, the acoustic performance of the coverings was very poor. In the range of 400–2500 Hz, they even produced a significant enhancement of the structural sound transmission, and the normalized impact sound pressure level, L_n_, was several decibels higher than for the bare M1 panel (Figure 12a). Consequently, the weighted values also slightly increased. The results were practically the same for each sample despite the different dynamic stiffness of the elastic layers. The high-frequency resonance of the M1 panel was distinctly marked at 2000 Hz in every case.

Heavy floating screeds consisted of a cement plate, 40 mm thick, positioned successively on: elastic polystyrene E-EPS 17/15, E-EPS 33/30, E-EPS 43/40, and mineral wool MW 30 mm. The floating screeds were significantly more effective, particularly at low and medium frequencies (Figure 12b). The high frequency resonance of bare M1 panel influenced the whole system and dramatically reduced the impact sound insulation around 2000 Hz. The L_n,w_ index was within the 71–74 dB range, and the highest value was obtained for the floating screed with the thinner layer of elastic polystyrene E-EPS 17/15.

The impact sound insulation of the M1 panel with examined top layers was obviously too low to satisfy acoustic requirements in residential buildings. On this account, the panel with the same screeds and floor coverings was additionally equipped with the C1x suspended ceiling.

**Figure 13:**
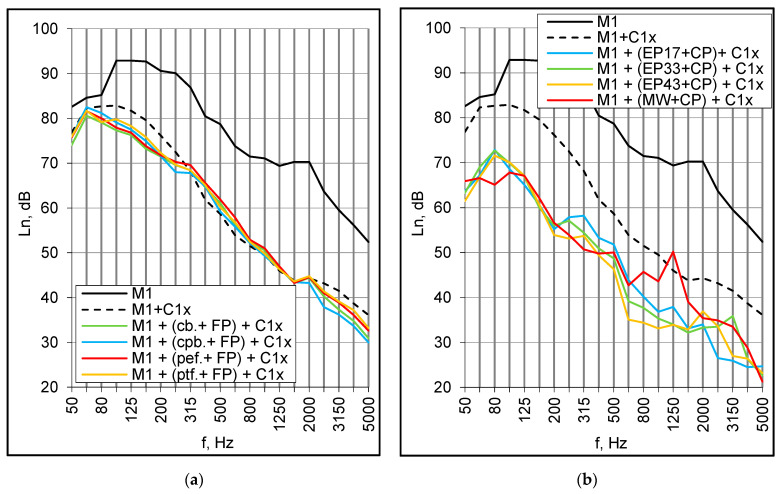
(**a**) M1 panel with floor coverings and suspended ceiling C1x; (**b**) M1 panels with floating screeds and suspended ceiling C1x.

The combination of floor coverings and the ceiling improved impact sound insulation by approximately 20 dB in the entire range except for the low-frequency area. The improvement, however, was evidently due to the installation of the ceiling itself, and the coverings had practically no influence on the results (Figure 13a). The values of L_n,w_ index were within the range of 66–67 dB, so they decreased by 18–19 dB relative to previously tested arrangements without the ceiling. Another layer of plasterboard (C2x) further improved the impact sound insulation by about 3 dB. However, the results were still not satisfactory and demonstrated that the typical floor coverings, even when combined with a suspended ceiling, were not adequate for composite floors designed for residential buildings.

The same series of measurements were repeated for the combinations of previously investigated heavy floating screeds and the ceiling (Figure 13b). The weighted normalized impact sound pressure level L_n,w_ dropped to 54 dB and was the same for all examined arrangements regardless of the type of the elastic underlayer. Adding the second plasterboard to the ceiling decreased the values by another 1–4 dB, so the performance level seemed promising.

### 4.2. Full-Scale Models

**Figure 14:**
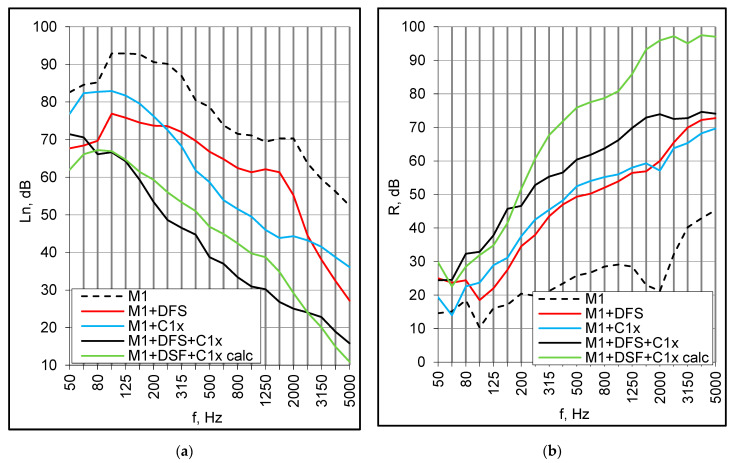
(**a**) Impact sound insulation: M1 panel with a dry floating screed (DFS) and suspended ceilings C1x. Measured and calculated (calc) values; (**b**) Airborne sound insulation: M1 panel with a dry floating screed (DFS) and suspended ceilings C1x. Measured and calculated (calc) values.

The preliminary study based on small samples demonstrated that the lightweight floor coverings, even when combined with a suspended ceiling, were insufficient to satisfy any acoustic requirements. Heavy floating screeds, on the other hand, behaved much better, but their execution is not practical for lightweight prefabricated buildings. Finally, a dry floating screed was recommended for further investigation. The screed consisted of a resilient underlayer made of mineral wool, 15 mm thickness, bound with the top layer of double 2 mm × 12.5 mm gypsum fiberboards. The M1 panel with the screed alone as well as the complete floor with the screed and suspended ceiling (single and double plasterboards), was examined. The coupled effect of the screed and ceiling was very optimistic in terms of both airborne and impact sound insulation (Figure 14a,b). The L_n,w_ index equaled 49–51 dB for a double and single ceiling, respectively. Airborne sound insulation of the complete system, expressed by the R_w_ + C index, ranged from 58 to 59 dB (Table 5).

The dry floating screed installed alone on the M1 panel improved impact sound insulation considerably in the entire frequency range. The suspended ceiling itself mitigated the high-frequency resonance and was even more effective, and the reduction of impact noise in the resonance area was 16–18 dB greater than produced by the screed (Figure 14a). However, these individual effects did not superpose. In the range of 200–2000 Hz, the measured values of normalized impact sound pressure level L_n,w_ of the complete floor were several decibels lower than resulting from the direct summation of respective individual improvements (Figure 14b). This proves that the floor components mutually interact, which makes any theoretical predictions more complicated.

In terms of airborne sound insulation, the effects of the screed and ceiling were comparable (Figure 14b). They also did not superpose, but the tendency was different than observed in the case of impact excitation. At low frequencies, both plots, i.e., measured and calculated from the individual improvements, ran parallel and were rather close. In the middle frequency area, the measured values were 5 to 16 dB lower. At high frequencies, the differences were even larger, ranging from 20 to 25 dB, but this probably resulted from the power shortage in the sending room. Nevertheless, the combined effect was not as profitable as might be concluded from the behavior of individual insulating layers.

## 5. Conclusions

Sound insulation of composite panels arranged in different configurations was investigated. The principal research question involved the development of a floor satisfying acoustic requirements in dwellings. The study showed that airborne sound insulation of stand-alone panels is poor and not acceptable for any application in residential buildings. The structural modifications, consisting in the alteration of the panels’ thickness, the surface mass of faces and core density and the application of a resilient interlayer separating the core from the faces, had a minor influence on the acoustic performance. Double set-ups with mineral wool in the cavity increased the sound insulation significantly at middle and high frequencies, whereas enlarged distance between panels was beneficial in the range of low and middle frequency. Nevertheless, the results demonstrated that the capability of double set-ups for further improvements is limited and the floor system should be equipped with additional insulating layers.

Impact sound insulation of stand-alone panels was very low. Several lightweight floor coverings were examined but they were inefficient and even produced local enhancement of the structural sound transmission. The floating floors were more effective and the combination of a dry floating screed and a suspended ceiling produced promising results in terms of both airborne and impact sound insulation. The results demonstrated that the complete floor system consisting of the load bearing composite elements and additional insulating layers achieves adequate acoustic properties and may be used in residential buildings. This gives a good prospect for general application of composite panels in housing and may stimulate further development of composite floor structures. The practical observations and conclusions may be useful for designers in modelling the floors, interpreting measurement results and validating numerical models. However, the results proved that the floor components mutually interacted, which complicates any theoretical predictions. Further investigations should concentrate on the technical solutions of the floating screed and suspended ceiling adjusted to the specific structure of composite panels. In addition, the question of low-frequency behavior should be examined more extensively.

## Figures and Tables

**Table 1:** The technical characteristics of composite panels include small samples (m1, m2, m3, m4) and full-scale models (M1, M2, M3).

Panel	Surface Mass	Total Thickness	Core Density	Surface Mass of a Single Face
	kg/m^2^	mm	kg/m^3^	kg/m^2^
m1/M1	9.7	60	40	3.8
m2/M2	11.6	35	40	5.3
m3/M3	10.1	35	70	4.1
m4	12.1	35	40	5.5

**Table 2:** Single number values for standalone composite panels, small samples (1390 mm × 2450 mm) and full-scale models (4190 mm × 2720 mm).

Small Samples		Full-Scale Models	
	R_w_ (C; C_tr_) dB		R_w_ (C; C_tr_) dB
m1	27 (−2; −3)	M1	27 (−2; −4)
m2	29 (−2; −2)	M2	28 (−2; −3)
m3	28 (−1; −3)	M3	28 (−1; −4)
m4	30 (−2; −3)	-	-

**Table 3:** Single number values for double set-ups, small samples (1390 mm × 2450 mm) and full-scale models (4190 mm × 2720 mm).

Component Panels	Empty Cavity 30 mm R_w_ (C; C_tr_) dB	Cavity 30 mm with Mineral Wool (MW) R_w_ (C; C_tr_) dB	Cavity 100 mm with Mineral Wool (MW) R_w_ (C; C_tr_) dB
m1-m1	39 (−1; −4)	42 (−4; −9)	52 (−4; −10)
m2-m2	44 (−3; −7)	49 (−4; −10)	57 (−4; −10)
M2-M2	31 (−1; −5)	40 (−7; −16)	49 (−7; −15)
M3-M3	33 (−2; −6)	39 (−6; −13)	48 (−6; −14)
m2-m3	40 (−2; −7)	44 (−5; −11)	52 (−4; −10)
m4-m4	41 (−3; −7)	51 (−4; −9)	56 (−2; −8)

**Table 4:** Single number values of M1 panel with additional insulating layers.

Floor Configuration	R_w_ (C; C_tr_), dB
M1 with a suspended ceiling C1x	51 (−3; −10)
M1 with a suspended ceiling C2x	54 (−3; −10)
M1 with a top insulating layer T1x	51 (−6; −14)

**Table 5:** Single number values for the M1 panel with a dry floating screed (DFS) and a suspended ceiling.

The Structure of the Floor System	L_n,w_, dB	R_w_ (C; C_tr_), dB
M1	84	27 (−2; −4)
M1 with a dry floating screed (DFS)	68	47 (−4; −11)
M1 with a dry floating screed (DFS) and suspended ceiling (C1x)	51	61 (−3; −10)
M1 with a dry floating screed (DFS) and suspended ceiling (C2x)	49	61 (−2; −7)

## Data Availability

The data presented in this study are available on request from the corresponding author. The data are not publicly available due to privacy restrictions.
